# Unveiling the Mental Health of Postpartum Women During and After COVID-19: Analysis of Two Population-Based National Maternity Surveys in Romania (2020–2025)

**DOI:** 10.3390/healthcare13080911

**Published:** 2025-04-16

**Authors:** Livia Ciolac, Dumitru-Răzvan Nițu, Elena Silvia Bernad, Adrian Gluhovschi, Daian-Ionel Popa, Teodora Toc, Anca Tudor, Anca-Laura Maghiari, Marius Lucian Craina

**Affiliations:** 1 Doctoral School, Faculty of General Medicine, “Victor Babes” University of Medicine and Pharmacy, 300041 Timisoara, Romania; livia.ciolac@umft.ro (L.C.); daian-ionel.popa@umft.ro (D.-I.P.); teodora.toc@umft.ro (T.T.); 2 Department of Obstetrics and Gynecology, Faculty of Medicine, “Victor Babes” University of Medicine and Pharmacy, 300041 Timisoara, Romania; nitu.dumitru@umft.ro (D.-R.N.); bernad.elena@umft.ro (E.S.B.); gluhovschi.adrian@umft.ro (A.G.); mariuscraina@umft.ro (M.L.C.); 3 Ist Clinic of Obstetrics and Gynecology, “Pius Brinzeu” County Clinical Emergency Hospital, 300723 Timisoara, Romania; 4 Center for Laparoscopy, Laparoscopic Surgery and In Vitro Fertilization, “Victor Babes” University of Medicine and Pharmacy, 300041 Timisoara, Romania; 5 Research Center for Medical Communication, “Victor Babes” University of Medicine and Pharmacy, 300041 Timisoara, Romania; 6 Department of Biostatistics and Medical Informatics, “Victor Babeș” University of Medicine and Pharmacy, 300041 Timisoara, Romania; 7 Department I—Discipline of Anatomy and Embryology, Faculty of Medicine, “Victor Babeş” University of Medicine and Pharmacy, 300041 Timisoara, Romania; boscu.anca@umft.ro

**Keywords:** perinatal mental health, postpartum depression, COVID-19 pandemic, post-pandemic period, EPDS, prevalence, screening

## Abstract

(1) **Background:** The COVID-19 pandemic caused widespread upheaval, presenting unique challenges for pregnant and postpartum women, who were already in a particularly vulnerable phase. As the COVID-19 pandemic and its public health response unfolded, it became crucial for clinicians and researchers to explore postpartum depression within the context of a global crisis. (2) **Methods:** We used data from two cross-sectional surveys of postnatal women conducted in our tertiary academic public hospital during the SARS-CoV-2 pandemic and the post-pandemic period, based on the retrospective assessments of two samples of mothers, each including 860 postpartum women. Our research has been conducted with the scope of evaluating postpartum depression disorder during and after the COVID-19 pandemic by using comparable data across time. (3) **Results:** The prevalence of postpartum depression was significantly higher among women who gave birth during the COVID-19 pandemic (major postpartum depressive disorder: 54.19%, minor depressive disorder: 15.58%), compared to pre-pandemic rates (10% in developed countries and 21–26% in developing countries) and post-pandemic rates (major depressive disorder 10.12%, minor depressive disorder 10.93%). The results of our research indicate that the COVID-19 pandemic had a major negative impact on perinatal mental health and, moreover, might have sped up an existing trend of the increasing prevalence of postpartum depression, despite the fact that the risk factors for postpartum depression disease remained consistent before, during, and after the pandemic. (4) **Conclusions:** Strengthening support systems during periods of heightened risk, such as during a pandemic, is crucial; therefore, policymakers and health planners should prioritize the mental health of this vulnerable group during global health crises or natural disasters, ensuring the implementation of effective mental health screenings, identification, enhanced support, follow-up, and reassurance measures to better address the challenges faced by susceptible postpartum women in future similar situations.

## 1. Introduction

Depression is among the most disabling conditions affecting women of reproductive age. Globally, for women aged 15 to 44 years, it represents the second leading cause of total disability, following HIV/AIDS [1]. Perinatal depression is considered a mood disorder characterized by the onset of depressive symptoms during pregnancy or within the first year postpartum. According to the DSM-5-TR, postpartum depression is encompassed within the broader classification of perinatal depression [2]. This term encompasses both antenatal (prenatal) and postnatal (postpartum) depressive episodes [2]. Perinatal depression affects approximately 6.5% to 20% of mothers globally [3]. Various studies have identified differing risk factors for perinatal depression, leading to limited consistency and comparability across findings [4]. The pathogenesis of perinatal depression remains incompletely understood; however, it is presumed to be multifactorial in nature [3]. Genetic predisposition, hormonal fluctuations, psychological vulnerability, and exposure to psychosocial stressors have all been proposed as contributing factors in its development [5]. A systematic review comprising 291 studies across 56 countries reported a global pooled prevalence of postpartum depression (PPD) of 17.7% (95% CI: 16.6–18.8%), underscoring the importance of recognizing and addressing PPD as a significant public health issue [6].

Postpartum depression (PPD), diagnosed when a new mother meets the DSM-5 criteria for a Major Depressive Episode in the first year after childbirth, is a significant concern both for individuals and public health. Biological mothers are affected in terms of their physical and psychological health, social relationships, and overall quality of life; meanwhile, children may face long-term impacts on their emotional and cognitive development [7]. Therefore, understanding the potential factors that influence the onset of postpartum depression is crucial, not only for the well-being of patients, their children or their families, but also for the healthcare system as a whole.

The widespread upheaval caused by the COVID-19 pandemic created unique challenges for pregnant and postpartum women, a group already navigating a particularly vulnerable period. Pregnant and postpartum women were regarded as a high-risk group for severe COVID-19 infection and symptoms. Uncertainty around the potential impact of COVID-19 on their babies, the safety of vaccines during pregnancy, and societal lockdowns, led to significant worry and stress among women who were pregnant or had recently given birth [8,9,10]. Furthermore, since the onset of the COVID-19 pandemic in Romania, in late February 2020, maternity services were reorganized into COVID support hospitals for cases diagnosed positively with the COVID-19 virus, birth partners (spouses or chosen support persons) were excluded from appointments and even the delivery itself, and mothers and babies were tested for the COVID-19 virus and separated in the hospital until the negative results came for both; also, addressability and perinatal support from both medical professionals were drastically reduced as long as the population was advised not to attend hospital unless strictly necessary [11,12,13]. A comprehensive multisystem analysis of the COVID-19 pandemic’s effects from 2021 revealed increases in various perinatal maternal psychosocial factors, including anxiety, postnatal depression, trauma, dissociation, and emotional well-being [14]. The review also highlighted significant increases in the rates of cesarean sections, stillbirths, ectopic pregnancies, hypertension, birth weight issues, and gestational diabetes [14]. When assessing the association between postpartum depression and the COVID-19 pandemic in a targeted manner, early studies revealed incongruent results. One study conducted between February and July 2020 in the United States, aimed to describe postpartum depression and its associated risk factors, found that one in three participants who gave birth during the pandemic experienced heightened postpartum depressive symptoms, particularly if they encountered specific risk factors [15].

As the COVID-19 pandemic and its public health response progressed, it became essential for clinicians and researchers to understand postpartum depression in the context of a global crisis. Peripartum patients during the pandemic likely had different experiences compared to those who gave birth before it [15]. Therefore, understanding the impact it may have had on post-pandemic time and identifying the risk factors for postpartum depression during this unprecedented pandemic period can help shape clinical practice and inform policy in the case of a similar event in the future.

Few studies have been conducted with the scope of evaluating postpartum depression disorder before, during, and after the COVID-19 pandemic by using comparable data across time; therefore, this is our research objective. We hypothesize that the prevalence of postpartum depression will be significantly higher among women who gave birth during the COVID-19 pandemic, compared to pre-pandemic or post-pandemic rates; hence, we aim to demonstrate the negative impact of the pandemic on perinatal mental health. We collected data from a national maternity survey, which included a sample of postpartum mothers from Romania, to compare the prevalence and also to identify individual factors that increased the risk of postpartum depression for new mothers during and after the pandemic.

The results of our research indicate that the COVID-19 pandemic had a major negative impact on the mental health of women in the perinatal period and, moreover, might have sped up an existing trend of the increasing prevalence of postpartum depression, despite the fact that the risk factors for postpartum depression disease remained consistent both before, during, and after the pandemic. Timely screening, identification, intervention, and follow-up of postpartum mothers are crucial for supporting women at risk. It is vital to strengthen support mechanisms during periods of increased risk, such as a pandemic.

## 2. Materials and Methods

### 2.1. Study Design and Sample Description

We used data from the cross-sectional surveys of postnatal women conducted in our tertiary academic public hospital from 1 March 2020 to 1 March 2023 (during the SARS-CoV-2 pandemic) and from 2 March 2023 to 2 March 2025 (post-pandemic), based on the retrospective assessments of two samples of mothers, each including 860 postpartum women, to compare the prevalence of postpartum depression during and after the pandemic, while assessing the association between several risk factors and postnatal depressive illness. Women were invited to take part in our research in the first year after they had given birth.

The manuscript of this observational study was prepared in accordance with STROBE guidelines. We conducted a G*Power (version 3.1.9.7) [16] analysis for the Chi-square test family, specifically for contingency tables as goodness-of-fit tests, using a power of 90%, a significance level of 0.05, one degree of freedom, and an effect size of 0.11. The estimated required sample size was 853 respondents, for each sample, from the pandemic and post-pandemic period.

For our study, patients were carefully screened and selected based on a detailed set of inclusion and exclusion criteria, aiming to ensure the specificity and consistency of the participant pool. A total of 1720 postpartum women (860 mothers from the pandemic period and another 860 mothers from the post-pandemic period) were eligible participants who were enrolled in this study. The observational study was conducted at the University Clinic of Obstetrics and Gynecology within the Timis County Emergency Clinical Hospital “Pius Brînzeu” in Timisoara, Romania, which is affiliated with the “Victor Babes” University of Medicine and Pharmacy.

Inclusion and Exclusion Criteria:

Participants were included in the study if they met the following criteria:

- Mothers aged 18–50 years, who gave birth during the SARS-CoV-2 pandemic or the post-pandemic period;
- Women who had given birth within the year prior to completing the survey;
- No history of psychiatric disorders;
- No history of peripartum depression in previous pregnancies;
- No past diagnosis or incidents of COVID-19 infection in the past year;
- Women who expressed interest in the topic and provided informed consent to participate in the study.

Participants were excluded from our study if they met any of the following exclusion criteria:

- Women currently using or with a history of psychotropic medication use;
- Women with high-risk pregnancies, including conditions such as preeclampsia, pregnancy-induced hypertension, gestational diabetes mellitus, intrauterine growth restriction, chronic diseases, chromosomal abnormalities, or known fetal anomalies;
- Women with a history of psychiatric disorders or mental health issues.

Informed consent was obtained from each participant prior to the study, as it involved sensitive data and vulnerable groups of individuals.

The Edinburgh Postnatal Depression Scale (EPDS) questionnaire [17] was used as the screening tool to assess the symptoms of postpartum depression disease. To better understand and highlight the characteristics of postpartum depression as well as the factors contributing to its development in the study participants, the following parameters were also considered: age, marital status, background, education level, working conditions (including occupational risks), socio-economic status, health status, personal medical history, parity, the method of conception, the type of delivery (as recommended by the medical consultant), the mother’s preferences regarding the mode of birth, the number of miscarriages, and the number of elective abortions.

### 2.2. Ethical Declarations

The Ethics Committee of the “Victor Babes” University of Medicine and Pharmacy in Timisoara, Romania, as well as the Ethics Committees of Timis County Emergency Clinical Hospital “Pius Brînzeu” in Timisoara, Romania, accepted the current study protocol on 1 March 2020, with the approval number 91. In accordance with ethical guidelines, all participants provided informed consent prior to participating in the research. This included their agreement to actively engage in the survey and consent to the collection of their health status and personal information, as it involved sensitive data and a vulnerable group of individuals. Additionally, this study implemented additional safeguards to protect the privacy and confidentiality of all participants. All collected data were thoroughly anonymized, with no personal identifiers linked to the information. This rigorous approach reinforced the study’s dedication to upholding the highest ethical standards throughout its duration.

### 2.3. Edinburgh Postnatal Depression Scale Questionnaire

Depressive disorder is a condition where psychometric assessment plays a crucial role in screening and confirming the diagnosis.

The questionnaire used to assess the symptoms of postnatal depression was the Edinburgh Postnatal Depression Scale (EPDS). The EPDS is the most commonly used standardized self-report tool for assessing postnatal depression. In 1987, Cox, Holden, and Sagovsky developed and tested this self-report tool in health centers in Edinburgh and Livingston (UK) to assist in identifying women who were experiencing postnatal depression [18]. Since its development, the EPDS has been validated against clinical diagnoses in over 37 languages and is considered the most widely used and well-validated screening tool for postpartum depression [19]. The EPDS has been validated across various countries and populations, including in Romania [20,21,22].

The questionnaire consists of 10 items that ask the women to rate the intensity of the depressive symptoms they have experienced over the past seven days [17]. Each item is rated on a four-point Likert scale (0–3), and the total score, ranging from 0 to 30, is obtained by summing the individual item scores [17]. A score greater than 10 indicates possible depression (minor depressive disorder), while a score of 13 or higher suggests major depressive disorder (moderate to severe) [17,23].

When compared to a clinical diagnostic interview, the EPDS exhibited the following psychometric properties: 78% specificity, 86% sensitivity, and a 73% positive predictive value for women scoring above 10 [24]. It is a straightforward tool that is easy to complete and interpret, requires no specialized psychiatric expertise, and can be easily integrated into healthcare services for all women during the postnatal period [17]. Validity studies indicate that the scale correctly identifies 92.3% of women with postpartum depression [24].

### 2.4. Statistical Assessment

JASPv0.19.3 software [25] was applied for statistical analyses. Quantitative variables were represented by the mean ± standard deviation or by the median (interquartile range), while nominal variables were represented by absolute frequencies and percentages. The Mann–Whitney U test was used to compare two independent series of numerical values that did not follow a normal distribution (the Shapiro–Wilk test was applied for normal distribution testing). The Chi^2^ Fisher’s Exact Test was used to analyze associations between categorical variables. A significance level of 0.05 was chosen for all tests; additionally, significant results were also described with 0.01 or 0.001 levels of significance. We conducted a G*Power analysis (version 3.1.9.7) [16] for the Chi-square test family, specifically for contingency tables as goodness-of-fit tests, using a power of 90%, a significance level of 0.05, one degree of freedom, and an effect size of 0.11. A small effect size was chosen because a weak association between the categorical variables was expected and a larger sample size was needed to detect such differences with a statistical power of 90% (higher than the commonly used 80%). This approach is appropriate in situations where even small differences are considered important.

The datasets from the two surveys were initially merged, and the weighted prevalence estimates of postpartum depression (defined as the percentage of women with a score greater than 10, which indicates a minor depressive disorder while a score of 13 or higher suggests a major depressive disorder) were calculated and compared for participants in both the pandemic and post-pandemic surveys. The distribution of socio-demographic characteristics, biopsychosocial factors, and obstetric indicators among the participants across the studies conducted during the COVID-19 pandemic as well as the post-pandemic period are presented using tables.

## 3. Results

### 3.1. Prevalence of Postpartum Depression in the 2020–2023 and 2023–2025 Surveys

This study involved two samples of women, each including 860 mothers in their first year after childbirth, one of which was conducted during the COVID-19 pandemic, and the second in the post-pandemic period.

The prevalence of postpartum depression was higher during the COVID-19 pandemic compared to the post-pandemic period. During the COVID-19 pandemic period, after assessing the clinical status of the women using the Edinburgh Postnatal Depression Scale (EPDS), 54.19% (466 women) were diagnosed with major depressive disorder, 15.58% (134 women) had minor depressive disorder, and 30.23% (260 women) showed no signs of depressive disorder. In the post-pandemic time frame, 10.12% (87 patients) had major depressive disorder, 10.93% (94 patients) had minor depressive disorder, while 78.95% (679 patients) had no depressive disorder.

Table 1 presents the weighted distributions of the socio-demographic characteristics and obstetric indicators for the women who participated in the surveys during the COVID-19 pandemic and the post-pandemic period.

**Figure 1:**
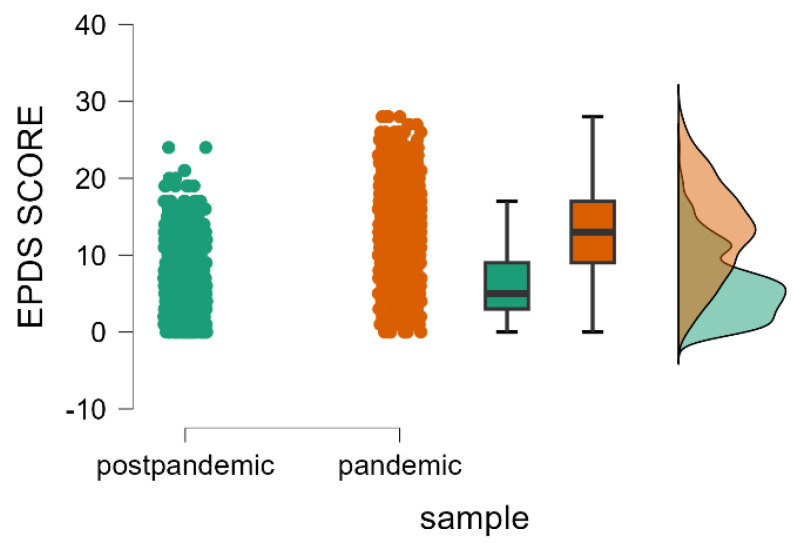
Distribution of the EPDS scores during the pandemic and post-pandemic periods, including individual data points, boxplots, and density plots.

We sought to analyze the prevalence of postpartum depression among mothers in the context of the pandemic of the coronavirus disease. Therefore, we found that the EPDS score was significantly increased during the pandemic period compared to the post-pandemic period (Mann–Whitney U test, *p* < 0.001) (Table 1 and Figure 1).

The proportion of mothers experiencing major (Chi^2^ Fisher’s Exact Test, *p* < 0.001) or minor (Chi^2^ Fisher’s Exact Test, *p* = 0.006) postpartum depressive disorder was significantly increased during the COVID-19 pandemic. Conversely, the proportion of mothers without postpartum depressive disorder was significantly higher in the post-pandemic period (Chi^2^ Fisher’s Exact Test, *p* < 0.001) (Table 1).

### 3.2. Factors Associated with Postnatal Depression

Within our analyses, there was evidence of a significant association between selected socio-demographic factors and obstetric indicators (maternal age, marital status, area of residence, level of education, workplace hazard, socio-economic conditions, health status, parity, method of achieving pregnancy, type of birth) and postpartum depression disease among the pandemic and post-pandemic surveys (Table 1).

**Figure 2:**
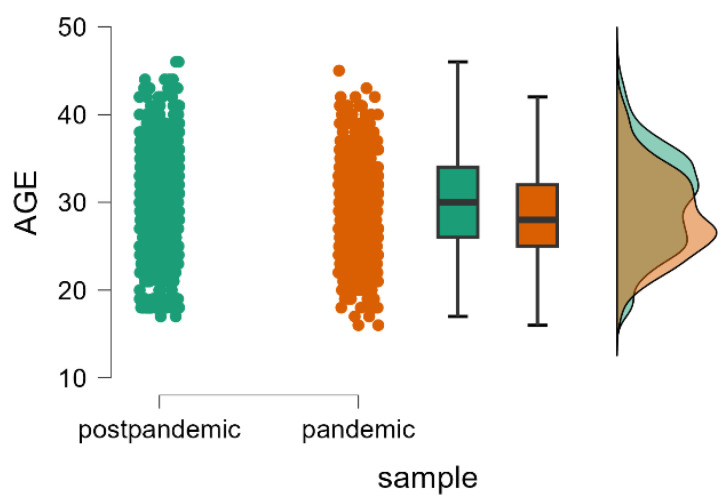
Distribution of the maternal age during the pandemic and post-pandemic periods, including individual data points, boxplots, and density plots.

The mean value of the maternal age was significantly higher in the post-pandemic sample compared to the pandemic sample (Mann–Whitney U test, *p* < 0.001), indicating that advanced age may serve as a protective factor against the onset of postpartum depression. (Table 1 and Figure 2).

Marital status appeared to have been affected by the transition through the coronavirus disease pandemic. The proportion of divorced mothers was significantly higher in the post-pandemic period compared to the pandemic period (Chi^2^ Fisher’s Exact Test, *p* < 0.001) and, furthermore, the proportion of single mothers was also significantly greater in the post-pandemic period, compared to the pandemic period (Chi^2^ Fisher’s Exact Test, *p* < 0.001). On the other hand, the proportion of married women was significantly increased during the pandemic period, compared to the post-pandemic period (Chi^2^ Fisher’s Exact Test, *p* = 0.005).

Urban areas, considered high-incidence zones for COVID-19, seemed to be a significant independent factor contributing to the occurrence of postpartum depression during the pandemic. The proportion of mothers from urban backgrounds was significantly increased during the pandemic period, compared to the post-pandemic period (Chi^2^ Fisher’s Exact Test, *p* < 0.001).

The increased level of education did not constitute a protective factor against developing postnatal depression during the coronavirus pandemic. Although the proportion of mothers with higher education was significantly higher during the pandemic period compared to the post-pandemic period (Chi^2^ Fisher’s Exact Test, *p* = 0.003), the prevalence of postpartum depressive disorder was higher during the COVID-19 pandemic. In contrast, the proportion of women with primary and vocational education was significantly higher in the post-pandemic period compared to the pandemic period (Chi^2^ Fisher’s Exact Test, *p* < 0.001).

Workplace hazards may have served as an additional risk factor for the development of depressive disorders during the postpartum period. The proportion of women working in high-risk environments was significantly higher during the pandemic period compared to the post-pandemic period (Chi^2^ Fisher’s Exact Test, *p* = 0.002). Additionally, the proportion of women working in medium-risk environments was significantly higher during the pandemic period compared to the post-pandemic period (Chi^2^ Fisher’s Exact Test, *p* < 0.001). In contrast, the proportion of women working in low-risk environments was significantly greater in the post-pandemic period than in the pandemic period (Chi^2^ Fisher’s Exact Test, *p* < 0.001), with the low risk at the workplace acting as a protective factor.

The proportion of mothers living in very good socio-economic conditions significantly increased in the post-pandemic period compared to the pandemic period (Chi^2^ Fisher’s Exact Test, *p* < 0.001). Conversely, the proportion of mothers in poor socio-economic conditions significantly rose during the pandemic period compared to the post-pandemic period (Chi^2^ Fisher’s Exact Test, *p* < 0.001). Furthermore, the proportion of women living in satisfactory socio-economic conditions was significantly higher in the pandemic period compared to the post-pandemic period (Chi^2^ Fisher’s Exact Test, *p* = 0.027). Living in poor or satisfactory socio-economic conditions at the time of the coronavirus pandemic seemed to create a favorable environment for the occurrence of postnatal depression.

The proportion of postpartum women reporting good health significantly increased in the post-pandemic period compared to the pandemic period (Chi^2^ Fisher’s Exact Test, *p* < 0.001), hence the mother’s good health status can be considered a protective factor. In contrast, the proportion of women with poor health significantly rose during the pandemic period compared to the post-pandemic period (Chi^2^ Fisher’s Exact Test, *p* = 0.023). Additionally, the proportion of women with a satisfactory health status was significantly higher in the pandemic period compared to the post-pandemic period (Chi^2^ Fisher’s Exact Test, *p* < 0.001). Poor or fair health status appeared to be an additional risk factor for the development of postpartum depression during the pandemic.

Regarding the type of birth, the proportion of mothers who gave birth naturally was significantly increased during the pandemic period, compared to the post-pandemic period (Chi^2^ Fisher’s Exact Test, *p* = 0.016).

The COVID-19 pandemic had a negative impact on fertility-related stress, which in turn has affected fertility and early pregnancy outcomes. The proportion of women who became pregnant following treatment was significantly increased in the pandemic period, compared to the post-pandemic period (Chi^2^ Fisher’s Exact Test, *p* < 0.001), while the proportion of those who became pregnant naturally was significantly increased in the post-pandemic period, compared to the pandemic period (Chi^2^ Fisher’s Exact Test, *p* < 0.001).

The proportion of multiparous women was not significantly increased in the post-pandemic period, compared to the pandemic period (Chi^2^ Fisher’s Exact Test, *p* = 0.809), parity did not appear to be influenced by the pandemic/post-pandemic period.

Table 2 highlights the fact that during the COVID-19 pandemic, the occurrence of postpartum depressive disorder was significantly influenced by the type of delivery (with vaginal birth potentially acting as a protective factor, Chi-square, *p* = 0.0012), education level (higher education appearing to offer protection, Chi-square, *p* = 0.028), and health status (a higher proportion of mothers with postpartum depression was observed among those reporting fair or good health, Chi-square, *p* < 0.001) [26].

In the case of the patients from the post-pandemic group, a significant association was established between the presence/absence of postpartum depression and the level of education (Chi^2^ Fisher’s Exact Test, *p* < 0.001). Specifically, the proportion of patients with middle schooling was significantly higher among those who experienced postpartum depression (Chi^2^ Fisher’s Exact Test, *p* < 0.001).

Likewise, a significant association was also found between socio-economic conditions and the presence or absence of postpartum depression (Chi^2^ Fisher’s Exact Test, *p* = 0.004). Thus, the proportion of mothers with satisfactory socio-economic conditions was significantly higher among those who experienced postpartum depression (Chi^2^ Fisher’s Exact Test, *p* = 0.002).

Table 3 highlights the fact that a significant association was established between socio-economic conditions and the type of birth (Chi^2^ Fisher’s Exact Test, *p* = 0.002). Thus, the proportion of mothers with a good socio-economic status was significantly increased among those who gave birth by cesarean section (Chi^2^ Fisher’s Exact Test, *p* < 0.001), while the proportion of those with very good and satisfactory socio-economic conditions was significantly increased among those who gave birth vaginally (Chi^2^ Fisher’s Exact Test, *p* = 0.036, respectively *p* = 0.019).

In the case of mothers from the post-pandemic sample, the association between the type of birth and the mother’s level of education was significant (Chi^2^ Fisher’s Exact Test, *p* = 0.019). Specifically, the proportion of patients with a primary school education was significantly higher among those who had a natural birth (Chi^2^ Fisher’s Exact Test, *p* = 0.004).

## 4. Discussion

### 4.1. Implications and Interpretation of Research Findings

There is increasing evidence that highlights the negative impact of giving birth during a pandemic period on women’s mental health. Multiple systematic reviews and meta-analyses have reported the prevalence of postpartum depression during the COVID-19 pandemic, with pooled estimates varying between 17% and 34% [14,27,28,29,30]. Most of these reviews have found higher rates of postpartum depression during the pandemic [14,27,28,29,30], compared to pre-pandemic levels (global pooled prevalence of 17–18%) [6,31]. The research papers conducted before and during the COVID-19 pandemic vary widely in terms of the target population sample, mental health measures, and sampling methods. This heterogeneity makes it challenging to reliably pool and compare prevalence estimates, complicating the evaluation of the pandemic’s impact on postpartum depression. Just a few studies have examined the impact of the COVID-19 pandemic on postpartum depression by using comparable data over time, and to date, there are no such studies that have been conducted in Romania. Our research aimed to explore the link between postpartum depressive disorder and the COVID-19 pandemic by comparing data from the pandemic and post-pandemic periods, while also exploring the influencing factors.

It is likely that women already experiencing physical or mental health conditions, such as postpartum depression, are likely to be the most negatively impacted by a pandemic [32]. Many studies examining the impact of the COVID-19 pandemic postpartum depression have yielded conflicting results. Some studies have shown that the prevalence of postpartum depression remained unchanged or even decreased during the COVID-19 pandemic [33,34,35], while others have reported an increase in prevalence [36,37,38].

### 4.2. Summary of Main Findings

The results of our cross-sectional survey, conducted during the SARS-CoV-2 pandemic and based on a retrospective evaluation of 860 postpartum women, revealed that the prevalence of major postpartum depressive disorder was 54.19% (466 women), 15.58% (134 women) experienced minor depressive disorder, while 30.23% (260 women) showed no signs of depressive disorder within the first year after delivery [26]. When comparing these results with data from studies conducted prior to the pandemic, there was a concerning increase in the prevalence of postpartum depression. In the pre-pandemic period, the global incidence of postpartum depressive disorder was approximately 10% in developed countries and 21–26% in developing countries [39,40]. Our survey conducted in the post-pandemic phase found that 10.12% (87 women) experienced major depressive disorder, 10.93% (94 women) had minor depressive disorder, and 78.95% (679 women) reported no depressive disorder during the postpartum period. When comparing these results with those from studies conducted before the pandemic, there was a concerning increase in the prevalence of postpartum depression during the SARS-CoV-2 pandemic, followed by a notable decrease in the post-pandemic period, which seems both natural and expected. It is important to emphasize that one of the inclusion criteria for participation in our study was the absence of a history of peripartum depression in previous pregnancies, as prior peripartum depression is a well-established predictor of future episodes, independent of external factors. Including women with such a history could have introduced a significant confounding variable, thereby complicating the ability to isolate the specific impact of the pandemic context. The proportion of mothers experiencing major or minor postpartum depressive disorder was significantly higher during the COVID-19 pandemic; in contrast, the proportion of mothers without postpartum depressive disorder was notably higher in the post-pandemic period. Likewise, we found that the EPDS score was significantly higher during the pandemic period compared to the post-pandemic period.

The COVID-19 pandemic represented a major global stressor. Previous research has also shown that during natural disasters, the prevalence of mental disorders among postpartum women was significantly higher than that of the general population [27]. In Romania, implemented measures such as social isolation and quarantine were effective in mitigating the spread of viral transmission; however, they had detrimental effects on mental health, particularly during the postpartum period, a time when women are especially vulnerable. It was also suggested that several factors contributed, during the COVID-19 pandemic, to the heightened incidence of depressive symptoms among postpartum women in our country. These factors included decreased physical activity during pregnancy, feelings of isolation, job loss, apprehension regarding visits to healthcare facilities during the pandemic, lack of a support person during childbirth, changes to birth plans, concerns about being unable to receive family visits post-delivery, and limited access to childcare services [32]. Multiple studies have indicated that lockdowns and social distancing measures during the pandemic played a significant role in impacting women’s mental health during the postpartum period [41,42,43].

Peripartum depression is known to have long-term effects on both the mother and the infant; therefore, identifying the risk factors involved can help facilitate targeted screening and the development of intervention strategies to mitigate the long-term impact of other natural disasters or pandemics on the mental health of mothers and their infant development. Our analysis revealed a significant association between selected socio-demographic factors and obstetric indicators (such as maternal age, marital status, area of residence, education level, workplace hazard, socio-economic conditions, health status, parity, method of conception, and type of birth) and postpartum depression, in both the pandemic and post-pandemic surveys. The average maternal age was significantly higher in the post-pandemic sample compared to the pandemic sample, suggesting that advanced age may act as a protective factor against the onset of postpartum depression. Also, a study of the specialized literature identified several maternal and perinatal factors associated with the development of PDS including a younger maternal age and being primiparous [44]. As women age, they may experience greater stability in their careers, finances, relationships, and mental health [44]. Our study, conducted in the pandemic period, showed that postpartum depression was influenced by parity [26]. Multiparous mothers were less likely to experience postpartum depression compared to primiparous mothers, with a study in the peer-reviewed literature suggesting that 50–60% of women experience postpartum depression after their first birth [45]. However, the proportion of multiparous women was not significantly increased in the post-pandemic period compared to the pandemic period, suggesting that parity was not influenced by the pandemic or post-pandemic period. Other factors including a younger age and being a first-time mother may lead to increased anxiety about delivery and motherhood [46].

Marital status seemed to have been influenced by the transition through the COVID-19 pandemic. The COVID-19 pandemic may have impacted family relationships in a negative way, as the proportion of divorced mothers was significantly higher in the post-pandemic period compared to the pandemic period, and the proportion of single mothers was also notably greater in the post-pandemic period. In contrast, the proportion of married women was significantly higher during the pandemic period than in the post-pandemic period. The results indicated a notable increase in the proportion of postpartum women who were single at the time of data collection, rising from 1.86% during the pandemic period to 5.12% in the post-pandemic period. While the decline in the proportion of married women and the increase in divorced women are plausible consequences of social stressors during and after the pandemic, the sharp rise in single mothers within such a short timeframe is more challenging to interpret. This could be attributed to shifting family structures, changing patterns in the reproductive decision-making process or methodological aspects (including differences in reporting, classification, or sample composition).

Urban areas, identified as high-incidence zones for COVID-19, appeared to be a significant independent factor contributing to the rise in postpartum depression during the pandemic, as the proportion of mothers from urban backgrounds was notably higher during the pandemic compared to the post-pandemic period. In turn, women in urban areas experienced greater social isolation, which may be linked to the preventive measures implemented, including quarantine, home isolation, and social distancing, designed to prevent the further spread of the virus; therefore, social isolation was proven as a significant predictor of postpartum depression among the urban women.

A higher level of education did not serve as a protective factor against postpartum depression during the COVID-19 pandemic. Despite a significantly higher proportion of mothers with higher education during the pandemic compared to the post-pandemic period, the prevalence of postpartum depressive disorder was still greater during the pandemic. A study aimed to evaluate the relationship between education level and the various symptoms of postpartum depression found that a lower education level was associated with more severe depressive symptoms; in contrast, a higher education level could be considered a stable indicator of socio-economic status for mothers of childbearing age [47]. This discrepancy is likely linked to the impacts of the COVID-19 pandemic circumstances.

Workplace hazards may contribute as an additional risk factor for developing depressive disorders during the postpartum period. The proportion of women working in high or medium-risk environments was significantly higher during the pandemic compared to the post-pandemic period. Conversely, the proportion of women working in low-risk environments was notably greater in the post-pandemic period, with the low risk at the workplace acting as a protective factor.

Living in poor or inadequate socio-economic conditions during the coronavirus pandemic appeared to create a conducive environment for the development of postnatal depression. The proportion of mothers living in poor or satisfactory socio-economic conditions significantly increased during the pandemic compared to the post-pandemic period, while the proportion of those living in very good socio-economic conditions notably rose in the post-pandemic period. Low socio-economic status, including factors like low income and education, as well as poor quality of life and limited access to mental health services, may contribute to the development of mental illness in women [48,49,50].

The proportion of postpartum women reporting good health significantly increased in the post-pandemic period compared to the pandemic period, suggesting that good health status could serve as a protective factor. In contrast, the proportion of women reporting poor or satisfactory health rose significantly during the pandemic compared to the post-pandemic period, with poor or satisfactory health status acting as an additional risk factor for the development of postpartum depression during the pandemic. Similar to previous studies, younger women who faced health issues during pregnancy, delivery, or the postpartum period were more likely to experience postpartum depression [46], with this being emphasized by the unfavorable circumstances generated in the context of COVID-19 pandemic.

A statistically significant increase in the rate of spontaneous vaginal deliveries was observed during the pandemic period compared to the post-pandemic period. The results reported a statistically significant increase in the proportion of cesarean deliveries in the post-pandemic period compared to the pandemic period. The observed increase may reflect changes in hospital policies or delivery protocols following the pandemic, evolving maternal preferences for scheduled births or an increase in high-risk pregnancies resulting from delayed prenatal care during the pandemic. Additionally, the higher maternal age noted in the post-pandemic sample could have also contributed to the increased cesarean delivery rate. Contrary to the findings in our study, where the mode of delivery was not associated with an increased likelihood for the onset of depressive symptoms, several studies have suggested that cesarean section may elevate the risk of postpartum depression [51]. Concerning the mode of delivery, the notably high rate of cesarean sections among the women in this study warrants attention, even though in our research it did not seem to be an independent risk factor for the development of postpartum depression, particularly in light of the potential impacts of the COVID-19 pandemic on obstetric practices and patient management. In Romania, it is important to note that the national rate of cesarean sections has been on the rise in recent years, a trend that appears to be independent of the COVID-19 pandemic. In 2012, the national cesarean section rate in Romania was 41.2% of all births, and by 2017, the country had risen to second place in Europe, following Cyprus, in terms of this rate [52,53]. This elevated rate in our country is primarily attributable to maternal preference, with the majority of cesarean sections being performed on request in the absence of clear obstetric indications. Furthermore, many healthcare providers may recommend cesarean sections without a robust medical indication, potentially driven by concerns over malpractice liability [53]. The elevated rate of cesarean sections observed in our study, surpassing the national average, may be attributed to the fact that our institution is a tertiary care center, which frequently receives transfers from smaller facilities where performing cesarean sections is not feasible.

It is important to highlight the apparent stability in the proportion of women with a history of at least one elective abortion across the two periods, with the proportion at approximately 12% in both groups. While overall rates remained comparable, there appeared to be a slight decrease in the proportion of women reporting multiple elective abortions (two or more) in the post-pandemic period. Although this difference may not be statistically significant, it may indicate subtle shifts in reproductive behavior, access to healthcare services, or broader changes in health-system functioning in the aftermath of the pandemic.

Recurrent miscarriage is a recognized factor that can impact maternal emotional well-being and may be particularly relevant in the context of postpartum mental health. The proportion of women experiencing two miscarriages was significantly higher during the pandemic, while the proportion of those with three miscarriages increased significantly in the post-pandemic period. These trends may reflect a subtle increase in the number of spontaneous abortions during the pandemic, changes in reproductive health, delays in reproductive care, stress-related effects during the pandemic, or selection effects in the aftermath of the pandemic.

The COVID-19 pandemic negatively impacted fertility-related stress, which in turn affected fertility and early pregnancy outcomes. The proportion of women who became pregnant through treatment significantly increased during the pandemic compared to the post-pandemic period, while the proportion of those who became pregnant naturally was significantly higher in the post-pandemic period. During the COVID-19 pandemic, birth rates in most higher income countries initially declined briefly, then quickly recovered, however, no consistent trends emerged afterward until early 2022, when they unexpectedly dropped [54].

These findings suggest that the COVID-19 pandemic is an acute public health issue that demands continuous, comprehensive, and long-term health education. This approach is essential to effectively reduce panic and fear among women, enhancing their ability to respond to similar challenges in the future.

### 4.3. Strengths and Limitations

Our study had several strengths, along with a few limitations.

The strengths of this study included the large, population-based samples of women from both the pandemic and post-pandemic surveys, as well as the use of survey weights to enhance the representativeness of the samples and the validity of the prevalence estimates. The availability of all the comparative data from two consecutive surveys with consistent sampling frames during and after the COVID-19 pandemic enabled us to accurately assess the true impact of the pandemic on postpartum depression. Another strength was the inclusion of a standardized depression measure, which had a validated cut-off point, enabling comparison with other studies existing in the field. Ideally, postpartum depression should be evaluated through a structured clinical interview. However, given the resource-intensive and non-anonymous nature of such assessments, they are not practical for large observational research studies. Thus, it was recommended to use a valid and widely recognized self-assessment tool, such as the Edinburgh Postnatal Depression Scale (EPDS) questionnaire. This tool was employed in our research to screen for the symptoms of postpartum depression. Another strength of this study was the consistency in the timing of measurements across both surveys, as both surveys recruited women in their first year after childbirth.

Limitations include the cross-sectional design of the pandemic/post-pandemic surveys which made establishing causality challenging. Also, as a cross-sectional study, it did not facilitate the establishment of a clear causal relationship between postpartum depression and the factors associated with this disease. Although this was a region-based population study with two large sample sizes, our data were derived from a cross-sectional design, and the use of non-probability convenience sampling may have limited the generalizability of the results to all mothers. Future studies could explore alternative methods, such as prospective designs or other objective measures, to address these limitations. Since the study design was cross-sectional, we lacked baseline data on the pre-pandemic levels of postpartum depression risk. An ideal approach would have been to include a comparable group representative of the pre-pandemic period, in order to objectively analyze the evolution of postpartum depression prevalence over time and the impact of the COVID-19 pandemic within the Romanian population.

Furthermore, the use of the self-reporting Edinburgh Postnatal Depression Scale as a screening tool may have led participants to provide responses they deemed socially acceptable.

Socio-demographic factors, obstetric indicators, satisfaction with birth, and mental health before and during pregnancy may all be perceived more negatively through the lens of postpartum depression. There also were socio-demographic differences between the respondents. The risk factors which were included in the analysis were limited to those assessed in the surveys. Additional factors, identified in the literature as being associated with postpartum depression, were not included in either of the surveys. Future studies, whether cross-sectional or longitudinal, should incorporate these and other known factors (intimate partner violence, a family history of depression, previous childbirth experiences, family situation), as it is crucial to understand how they contribute to the risk of postpartum depression, both in normal circumstances and during times of crisis. Future research should also explore the mental health of women’s partners in this context, as well as the mental health of both women and their partners in the post-pandemic era.

These issues call for further research to connect women’s integrative and preventive health policies and programs, aiming to improve both reproductive health and maternal mental health [55,56,57]. Primary care providers, along with other healthcare professionals and policymakers in the reproductive and maternal health fields, must also give this issue their attention [58,59]. We strongly advocate for greater national focus on maternal mental health and encourage further research into the impact of perinatal mental health issues, such as postpartum depression, on the well-being of the mother, baby, family, society, and economy. The impact of a challenging birth experience (birth trauma) and the potential emotional effects of the intention to breastfeed, breastfeeding success, and their influence on perinatal mental health and overall maternal wellness also require further study.

## 5. Conclusions

The findings of this study aligned with the increasing body of evidence demonstrating the negative impact of COVID-19 on perinatal mental health. The prevalence of postpartum depression was markedly higher among women who gave birth during the COVID-19 pandemic (major postpartum depressive disorder 54.19%, minor depressive disorder 15.58%), compared to pre-pandemic rates (10% in developed countries and 21–26% in developing countries) and post-pandemic rates (major depressive disorder 10.12%, minor depressive disorder 10.93%). Therefore, the results indicated that the SARS-CoV-2 pandemic, particularly for giving birth during the peak of the pandemic, negatively affected mental health and may have contributed to the growing prevalence of postpartum depression. To our knowledge, this is the first population-based study in our country to provide comparable prevalence estimates of postpartum depression during and after the pandemic. Our analysis identified a significant relationship between various socio-demographic factors and obstetric indicators (such as maternal age, residence area, workplace hazards, socio-economic conditions, health status, parity, and conception method) and postpartum depression, in both surveys. More specifically, a younger maternal age, women from urban areas, being primiparous, working in high or medium-risk environments, living in poor or satisfactory socio-economic conditions, poor or satisfactory health status, and pregnancy obtained with previous treatment, had a higher risk for postpartum depression. In contrast, a higher level of education, marital status (married women), or the type of birth (spontaneous vaginal delivery) seemed to not serve as a protective factor against postpartum depression during the COVID-19 pandemic. These risk and protective factors remained consistent for women who gave birth both before and during the pandemic.

Hence, policymakers and health planners should prioritize the mental health of this vulnerable group of women during global health crises or natural disasters and do their best to implement effective mental health screening, stronger support, and reassurance measures to cope in the future with the impact of a similar situation in the case of susceptible postpartum women. Healthcare professionals should actively encourage new mothers to openly discuss their mental well-being and seek assistance for any symptoms of depression. Timely assessment, diagnosis, intervention, and follow-up are crucial for supporting women at risk or those experiencing symptoms. It is essential that mechanisms to detect and support women remain in place and also are strengthened and repeated in times of heightened risk, such as the pandemic. The NICE guidelines recommend that all women should be screened for their mental health concerns at six weeks postpartum [60].

## Figures and Tables

**Table 1:** Distribution of socio-demographic characteristics and obstetric indicators among study participants.

Association Variables		Sample		*p* Value
		COVID-19 Pandemic Period (n = 860)	Post-Pandemic Period (n = 860)	
Age Mean ± SD Median (Q1–Q3)		28.45 ± 5.18 28 (25–32)	30.06 ± 5.62 30 (26–34)	<0.001 *
EPDS score Mean ± SD Median (Q1–Q3)		13.06 ± 6.01 13 (9–17)	6.12 ± 4.56 5 (3–9)	<0.001 *
Postpartum depressive disorder n (%)	Major postpartum depressive disorder	466 (54.186%)	87 (10.116%)	<0.001 *
	Minor postpartum depressive disorder	134 (15.581%)	94 (10.930%)	0.006 *
	Without postpartum depressive disorder	260 (30.233%)	679 (78.954%)	<0.001 *
Marital status n (%)	Married	746 (86.744%)	703 (81.744%)	0.005 *
	Cohabiting	98 (11.395%)	89 (10.349%)	0.536
	Divorced	0 (0%)	24 (2.791%)	<0.001 *
	Single	16 (1.860%)	44 (5.116%)	<0.001 *
Area of residence n (%)	Urban	598 (69.535%)	468 (54.419%)	<0.001 *
	Rural	262 (30.465%)	392 (45.581%)	
Level of education n (%)	Higher education	509 (59.186%)	447 (51.977%)	0.003 *
	High school graduate	277 (32.209%)	266 (30.930%)	0.604
	Vocational school	0 (0%)	56 (6.512%)	<0.001 *
	Middle school	74 (8.605%)	55 (6.395%)	0.099
	Primary education	0 (0%)	36 (4.186%)	<0.001 *
Socio-economic conditions n (%)	Very good standard of living	167 (19.419%)	228 (26.512%)	<0.001 *
	Good standard of living	507 (58.953%)	503 (58.488%)	0.883
	Satisfactory conditions	160 (18.605%)	125 (14.535%)	0.027 *
	Poor living conditions	26 (3.023%)	4 (0.465%)	<0.001 *
Workplace hazard n (%)	High	72 (8.372%)	40 (4.651%)	0.002 *
	Medium	195 (22.674%)	139 (16.163%)	<0.001 *
	Low	593 (68.953%)	681 (79.186%)	<0.001 *
Health status n (%)	Good	683 (79.419%)	809 (94.070%)	<0.001 *
	Satisfactory	168 (19.535%)	51 (5.930%)	<0.001 *
	Poor	9 (1.047%)	0 (0%)	0.023 *
Parity n (%)	Primiparous	561 (65.232%)	417 (48.488%)	<0.001 *
	Secundiparous	242 (28.139%)	300 (34.883%)	0.003 *
	Tertiparous	45 (5.232%)	92 (10.697%)	<0.001 *
	Quadriparous	11 (1.279%)	30 (3.4883%)	0.005 *
	Multiparous (5+)	1 (0.116%)	21 (2.4418%)	<0.001 *
Number of miscarriages in their personal obstetric history n (%)	No miscarriage	689 (80.116%)	700 (81.395%)	0.534
	1 miscarriage	130 (15.116%)	117 (13.604%)	0.413
	2 miscarriages	34 (3.953%)	18 (2.093%)	0.030 *
	3 miscarriages	5 (0.581%)	19 (2.209%)	0.008 *
	4 miscarriages	2 (0.232%)	6 (0.697%)	0.235
Number of abortions performed upon request in their personal obstetric history n (%)	No abortion on request	750 (87.209%)	755 (87.790%)	0.766
	1 abortion on request	77 (8.953%)	83 (9.651%)	0.703
	2 abortions on request	23 (2.674%)	10 (1.162%)	0.032 *
	3 abortions on request	8 (0.930%)	8 (0.930%)	0.851
	4 abortions on request	0 (0%)	4 (0.465%)	0.130
	5 abortions on request	2 (0.232%)	0 (0%)	0.583
Method of achieving pregnancy n (%)	Naturally	824 (95.813%)	848 (98.604%)	<0.001 *
	In vitro fertilization	10 (1.162%)	8 (0.930%)	0.812
	With previous treatment	26 (3.023%)	4 (0.465%)	<0.001 *
Type of birth n (%)	Cesarean section	262 (30.465%)	392 (45.581%)	0.016 *
	Vaginal delivery	598 (69.534%)	468 (54.418%)	

*—significant difference.

**Table 2:** Comparative percentage representation of the cases by socio-demographic variables, health status, the type of birth, and the occurrence of depressive disorder during the COVID-19 pandemic [26].

Association Variables		Type of Birth		*p* Value	Postpartum Depression		*p* Value
		Cesarean Section	Vaginal Delivery		Absence	Presence	
Depressive disorder	Without	128 (25.80%)	132 (36.30%)	0.0012 *			
	Minor	78 (15.70%)	56 (15.40%)	0.98		------------	
	Major	290 (58.50%)	176 (48.40%)	0.0041 *			
Marital status	Married	441 (88.90%)	305 (83.80%)	0.0382 *	229 (88.10%)	517 (86.17%)	0.511
	Cohabiting	41 (8.30%)	57 (15.70%)	0.0011 *	26 (10.00%)	72 (12.00%)	0.465
	Single	14 (2.80%)	2 (0.50%)	0.026 *	5 (1.90%)	11 (1.83%)	0.837
Education level	Less than high school	24 (4.80%)	50 (13.70%)	<0.001 *	24 (9.20%)	50 (8.30%)	0.764
	High school graduate	160 (32.30%)	117 (32.10%)	0.991	67 (25.8%)	210 (35.00%)	0.010 *
	Higher education	312 (62.90%)	197 (54.10%)	0.012 *	169 (65.00%)	340 (56.70%)	0.028 *
Socio-economic conditions	Good	290 (58.50%)	217 (59.60%)	0.799	157 (60.40%)	350 (58.30%)	0.617
	Very good	111 (22.40%)	56 (15.40%)	0.013 *	60 (23.10%)	107 (17.80%)	0.087
	Poor	10 (2.00%)	16 (4.40%)	0.067	4 (1.50%)	22 (3.70%)	0.131
	Satisfactory	85 (17.10%)	75 (20.60%)	0.224	39 (15.00%)	121 (20.20%)	0.088
Health status	Good	386 (77.80%)	297 (81.60%)	0.202	232 (89.20%)	451 (75.20%)	<0.001 *
	Poor	6 (1.20%)	3 (0.80%)	0.816	1 (0.40%)	8 (1.30%)	0.404
	Fair	104 (21.00%)	64 (17.60%)	0.248	27 (10.40%)	141 (23.50%)	<0.001 *

*—significant difference.

**Table 3:** Comparative percentage representation of the cases by socio-demographic variables, health status, the type of birth, and the occurrence of depressive disorder in the post-pandemic period.

Association Variables		Type of Birth		*p* Value	Postpartum Depression		*p* Value
		Cesarean Section (545)	Vaginal Delivery (315)		Absence (679)	Presence (181)	
Depressive disorder	Without	428 (78.50%)	251 (79.70%)	0.104			
	Minor	54 (9.90%)	40 (12.70%)			------------	
	Major	63 (11.60%)	24 (7.60%)				
Marital status	Married	454 (83.30%)	249 (79.00%)	0.362	565 (83.20%)	138 (76.20%)	0.146
	Cohabiting	53 (9.70%)	36 (11.40%)		66 (9.70%)	23 (12.70%)	
	Divorced	12 (2.20%)	12 (3.8%)		18 (2.70%)	6 (3.30%)	
	Single	26 (4.80%)	18 (5.70%)		30 (4.40%)	14 (7.70%)	
Education level	Primary education	14 (2.60%)	22 (7.00%)	0.004 *	28 (4.10%)	8 (4.40%)	0.947
	Middle school	35 (6.4%)	20 (6.30%)	0.930	30 (4.40%)	25 (13.80%)	<0.001 *
	Vocational school	32 (5.9%)	24 (7.60%)	0.409	44 (6.50%)	12 (6.60%)	0.904
	High school graduate	168 (30.80%)	98 (31.10%)	0.988	218 (32.10%)	48 (26.50%)	0.174
	Higher education	296 (54.30%)	151 (47.90%)	0.082	359 (52.90%)	88 (48.60%)	0.345
Workplace Hazard	High	28 (5.10%)	12 (3.80%)	0.671	28 (4.10%)	12 (6.60%)	0.339
	Low	429 (78.70%)	252 (80.00%)		539 (79.40%)	142 (78.50%)	
	Medium	88 (16.10%)	51 (16.20%)		112 (16.50%)	27 (14.90%)	
Socio-economic conditions	Good	345 (63.30%)	158 (50.20%)	<0.001 *	404 (59.50%)	99 (54.70%)	0.280
	Very good	131 (24.00%)	97 (30.80%)	0.036 *	188 (27.70%)	40 (22.10%)	0.155
	Poor	2 (0.40%)	2 (0.60%)	0.917	2 (0.30%)	2 (1.10%)	0.431
	Satisfactory	67 (12.30%)	58 (18.40%)	0.019 *	85 (12.50%)	40 (22.10%)	0.002 *
Health status	Good	508 (93.20%)	301 (95.60%)	0.104	644 (94.80%)	165 (91.20%)	0.075
	Satisfactory	37 (6.80%)	14 (4.40%)		35 (5.20%)	16 (8.80%)	

*—significant difference.

## Data Availability

The original contributions presented in the study are included in the article; further inquiries can be directed to the corresponding author.

## Abbreviations

The following abbreviations are used in this manuscript:

PPD	Postpartum depression
EPDS	Edinburgh Postnatal Depression Scale
SARS-CoV-2	Severe acute respiratory syndrome coronavirus 2
DSM-5	The Diagnostic and Statistical Manual of Mental Disorders, Fifth Edition
DSM-5-TR	Diagnostic and Statistical Manual of Mental Disorders, Fifth Edition, Text Revision
HIV	Human Immunodeficiency Virus
AIDS	Acquired Immunodeficiency Syndrome
